# Microbial load in UTI, LRTI, BSI among acute ischemic stroke patients

**DOI:** 10.6026/973206300210339

**Published:** 2025-03-31

**Authors:** Shaik Mohiseen Begum, Amar Sajjan C., Afreen Iqbal

**Affiliations:** 1 Department of Microbiology Dr Patnam Mahender Reddy Institute of Medical Sciences, Chevella, Telangana, India

**Keywords:** Acute ischemic stroke, microorganisms, national institutes of health stroke scale (NIHSS), antibiotics, drug resistance

## Abstract

The incidence of urinary tract infections (UTIs), lower respiratory tract infections (LRTI) and blood stream infections in Acute Ischemic Stroke patients is of interest. Hence, a prospective cohort study was conducted on 60 acute ischemic stroke patients admitted within 48 hours from onset of stroke under department of Neurology, tertiary care hospital between Jan 2024 to July 2024. The mean age of the patients was 67.8 years. The average National Institutes of Health Stroke Scale (NIHSS) score was 10.2 ± 3.8 indicating moderate stroke severities. Samples from acute ischemic stroke (AIS) patients were were processed microbiologically for the presence of potential pathogens using standard microbial procedures and antibiotic susceptibility tests performed using disc diffusion technique. Production of AmpC Beta Lactamase, Carbapenemase and Methicillin resistant *Staphylococcus aureus* was detected by routinely used phenotypic methods. Most of the infection identified was UTI (48.3%) followed by lower respiratory tract infections 33.3% and the common microbial isolates were *E. coli* (33.3%) in majority of the acute stroke patients. Thus, regular screening for infections in stroke patients through clinical observation and microbiological testing (*e.g.*, cultures, blood tests) can help in early treatment and implementing best practices for rehabilitation, including mobility support, nutrition and skin care, can lower the risk of secondary infections.

## Background:

The microbiological profile of infections in acute ischemic stroke (AIS) patients is crucial in understanding the interplay between stroke and infection and its significance in public health lies in its impact on patient outcomes, healthcare burden and prevention strategies [1]. The management of infections in stroke patients demands more intensive resources (*e.g.*, antibiotics, critical care), contributing to the overall strain on healthcare systems, particularly in low-resource settings. The illness burden is higher in low-middle-income countries than in high-income ones and the highest prevalence of stroke was found in these nations [1]. Stroke is the second most prevalent cause of death worldwide, accounting for 13.7 million recorded fatalities annually. Over 60 years of age is now the average life expectancy in India [2-3], which has led to a rise in age-related, non-communicable diseases including stroke, which is now the country's fourth major cause of death and fifth leading cause of disability [4, 5, 6- 7]. The incidence of stroke in India was estimated to be between 105 and 152/100,000 individuals annually in a recent systematic analysis that mostly included cross-sectional data [8]. Ischemic and hemorrhagic strokes are the two main types. The incidence of stroke is significantly influenced by a number of risk factors, including obesity, hypertension, smoking, dyslipidaemia, diabetes mellitus, alcohol use, atrial fibrillation, carotid stenosis, inflammation. Approximately three-quarters of strokes are ischemic. Patients who have had a stroke are susceptible to a variety of microbial infections, frequently as a result of compromised immunity, hospitalization, or stroke-related sequelae [9- 10]. Although it is unclear how infectious agents contribute to stroke risk, there is some evidence that microorganism infection is associated with conditions including atherosclerotic plaques, metabolic imbalance, cardiovascular disease (CVD) and hypertension. The common bacterial pathogens causing infection in stroke patients include periodontal microflora, *Helicobacter pylori*, *Chlamydia pneumoniae*, *Haemophilus influenza*, *Mycoplasma pneumoniae*, *Mycobacterium tuberculosis*, *Streptococcus pneumonia*, *Coxiella burnetii*, *Staphylococcus aureus*, *Pseudomonas aeruginosa* *etc.* [11, 12, 13, 14, 15, 16, 17-18]. Through their virulence mechanisms, which include toxins, enzymes, interference with the host immune response and infectious endocarditis, pathogenic bacteria are among the infectious organisms that contribute most to the development of CVD [10]. The literature indicates that sepsis and endocarditis are the two primary underlying conditions that raise a person's risk of stroke. *Streptococcus pneumonia* (67%) is the most prevalent pathogen found in bacterial meningitis and encephalitis in stroke patients. Group B streptococci, *Staphylococcus aureus* (21%), Neisseria meningitides, *Pseudomonas aeruginosa*, and Listeria monocytogenes are the next most common pathogens. According to an analysis of a Spanish retrospective study, endocarditis caused by *Staphylococcus aureus* and vegetation size greater than 30 mm are risk variables associated with embolic activity in infective endocarditis [19]. About 26% of patients with tuberculous meningitis experienced an ischemic stroke as a neurovascular consequence, according to retrospective research conducted in Pakistan [20]. Previous studies demonstrated that iatrogenic, clinical and anatomical variables could predict and impact infection following a stroke [21]. Common side effects are in stroke victims, particularly those who are immobile or have dysphagia or trouble swallowing. When food or liquid gets into the lungs, it can lead to aspiration pneumonia. In addition to pneumonia, immobility or aspiration can result in other respiratory illnesses, such as bronchitis. Infections like cellulitis or abscesses can arise as a result of immobility and pressure on specific body parts (pressure ulcers or bedsores). Stroke patients may have a severe systemic infection, which frequently follows a secondary infection (*e.g.*, pneumonia, UTI, or skin infection) [21]. If not treated right away, sepsis can be fatal. Patients who have had a stroke are susceptible to Clostridium difficile infections, especially after using antibiotics. Infections of the central nervous system, such as meningitis or encephalitis, are uncommon but can occur in stroke patients due to compromised immune systems or problems following the stroke [21]. Therefore, it is of interest to determine the incidence of urinary tract infections (UTIs), lower respiratory tract infections (LRTI) and blood stream infections in acute ischemic stroke patients admitted in a tertiary care hospital and to isolate the predominant causative pathogens and identifying their antibiotic susceptibility pattern.

## Materials and Methods:

## Study design and study settings:

A prospective cohort study was conducted on 60 Acute Ischemic Stroke patients admitted within 48 hours from onset of stroke under department of Neurology, tertiary care hospital between Jan 2024 to July 2024.

## Inclusion criteria:

Patients presenting with an acute onset of focal neurological deficit combined with neuroimaging evidence of cerebral infarction by CT or MRI, Patients with symptoms of UTI, LRTI and blood stream after 48h of hospital admission.

## Exclusion criteria:

Patients with an intracranial hemorrhage, hypoglycemia or other causes of focal deficit and Patients with severe liver, renal and cardiac dysfunction.

## Methodology:

## Study population:

All the demographic data like age, gender, co-morbid conditions were collected from the medical records. For assessing the stroke, The National Institutes of Health Stroke Scale (NIHSS)-is frequently used as an early secondary outcome measure in stroke trials. It is usually assessed 24 hours or 5 to 7 days after the treatment. It measures neurological deficit rather than functional outcome. NIHSS scores range from 0 to 42, with higher scores indicating more severe neurological deficit.

## Specimen collection, Culture isolation and identification from UTI patients:

Clean catch midstream urine was collected from the UTI patients. In catheterized patients, urine aspirated from the catheter tube after clamping distally and disinfecting. After the collection, a wet mount examination was performed to identify the presence of pus cells, epithelial cells, RBCs and microorganisms. The samples were inoculated with calibrated loop onto sheep blood agar, chocolate agar and MacConkey agar plates and incubated at 37°C for 24 hrs. Kass concept was used to determine the significant bacterial count of urine sample and the significant bacteriuria is defined as a urine sample containing >105 cfu/ml of urine. The isolated organisms were identified by colony morphology, Gram staining, followed by standard biochemical reactions like catalase test, coagulase test, Oxidase test, bile aesculin hydrolysis, Lysine iron agar test, Indole test, Citrate utilization test, urease test, Triple sugar iron agar test, mannitol fermentation.

## Specimen collection, culture isolation and identification from LRTI patients:

From LRTIs patients' samples like sputum, tracheal aspirate, bronchoalveolar lavage (BAL), lung aspirate collected by transtracheal aspiration and pleural fluid collected by thoracocentesis were obtained. Direct Gram staining was performed from exudates for the presumptive identification of microorganisms and for the identification of pus cells and epithelial cells. The quality of sputum as checked and scoring was done by Barlett scoring system. Ziehl-Neelsen technique was used to identify the *Mycobacterium tuberculosis*. Specimens are inoculated onto blood agar, chocolate agar and MacConkey agar and incubated overnight. The organisms were identified based on their colony morphology and biochemical tests. In BSI patients, blood samples were collected for culture using a sterile syringe and they were collected in pairs from two separate venipunctures. 8-10 ml of blood per bottle was collected in adult bottle. Collected blood was dispensed directly into blood culture bottle at the bedside. The collected blood was inoculated into Castaneda's biphasic medium containing BHI agar slope and BHI broth. The blood was inoculated in the medium at a dilution of 1:5 for better dilution and anticoagulant sodium polyanethol sulfonate was added to the medium. The blood culture bottles were incubated in an upright position at 37°C for up to 7 days. The subcultures were made from the BHI broth onto blood agar and MacConkey agar.

## Antibiotic susceptibility pattern:

All bacterial isolates were subjected to Antibiotic sensitivity test by using standard "Disc diffusion method" (modified Kirby-Bauer method) on Mueller -Hinton agar (disc diffusion test as per the National committee for Clinical Laboratory Standards (NCCLS) recommendations. Interpretation of zone size into sensitive, intermediate or resistant is based on the standard zone size interpretation chart. The antibiotics were selected based on the CLSI (Clinical and laboratory standard institute) guidelines which provide a panel of antimicrobial agents appropriate for testing members of the Enterobacteriaceae, Pseudomonas and other Gram-negative and Gram-positive microorganisms. For Enterobacteriaceae, the antibiotics tested were Amoxy-clav (AMC, 20/10µg), Cefoxitin (CX, 30µg), Ceftazidime (CAZ, 30µg), Ceftazidime-clavulanic acid (CAC, 30µg), Cefotaxime (CTX, 30µg), Ceftriaxone (CTR, 30µg), Cefuroxime (CXM, 30µg), Cefipime (CPM, 30µg), Meropenem (MRP, 10µg/ml), Aztreonam (AT, 30µg/ml). For *Staphylococcus aureus* the antibiotics tested according to CLSI were Linezolid (LZ, 30µg), Vancomycin (VAN, 30µg), Cefoxitin (CX, 30µg), Penicillin (P, 10U), Amoxy-clav (AMC, 20/10µg), Gentamicin (GEN, 10µg), Cotrimoxazole (25µg), Clindamycin (2µg/ml). CLSI M100 edition was followed for antimicrobial agent breakpoints in disk diffusion method. Zone diameter values used to categorize an organism as susceptible, susceptible dose-dependent, intermediate or resistant. The size of this inhibition zone, measured in millimeters, reflects the bacteria's susceptibility to the antibiotic AmpC, ESBL, Carbapenemase and MRSA detection.

The production of AmpC was detected by phenotypic test like measuring the zone of inhibition size around the cefamycin drug like cefoxitin disc. If the zone size is less than 18mm it is positive for AmpC production and Extended spectrum beta-lactamase production (ESBL) production was tested with the CLSI confirmatory test using Ceftazidime (30 µg) disc alone and in combination with Clavulanic acid (10 µg). The test was considered positive when an increase in the growth-inhibitory zone around the CAZ disc with CA was 5 mm or greater of the diameter around the disk containing CAZ alone. The microorganisms which are resistant to meropenem antibiotic by showing the measured zone size of less than 23mm was considered as carbapenemase producer. The MRSA production was detected by measuring the zone size of less than 22mm surrounding cefoxitin.

## Results:

The study included a total of 60 patients and among them 38 males (63.3 %) and 22 were females (36.6%). The mean age of the patients was 67.8 years. In most of the study subjects of the current study, the major predisposing factors were Diabetes 46.7% followed by Hypertension 31.7% (Table 1) (Figure 1 - see PDF). The average NIHSS score was 10.2 ± 3.8 indicating moderate stroke severities. In majority of the acute stroke patients, most of the infection identified was UTI (48.3 %) followed by lower respiratory tract infections 33.3% (Table 2). Among the 60 samples received, only 46 samples were culture positive. The common microorganism isolated was E.coli (33.3%) followed by *Klebsiella pneumoniae* (16.6%), *Staphylococcus aureus* (8.3%) (Table 3). Among the three isolates of *A. baumanii*, most of the strains showed sensitivity to CZA, TE, PIT and AK. All the 3 strains were resistant to AMC and CX. Three strains were considered as AmpC producers as they showed sensitivity to cefoxitin less than 18mm. Among the three isolates, 2 were ESBL produces because they were resistant to all cephalosporin generations including aztreonam and two strains were carbapenemase producers as they show resistant to meropenem antibiotic less than 18mm according to CLSI guidelines (Table 4). Among the two isolates of *S. pneumoniae*, all the strains showed sensitivity to LVX, VAN, MRP, E, CD, DOX, CPM and LZ (Table 5). Among the 20 isolates of *E.coli*, most of the strains showed sensitivity to CZA, CX, PIT, CAC, AK and MRP. Among the 20 strains, 6 were considered as AmpC producers as they showed sensitivity to cefoxitin less than 18mm, 12 strains were ESBL produces because they were resistant to all cephalosporin generations including aztreonam and two strains were carbapenemase producers as they show resistant to meropenem antibiotic less than 23mm according to CLSI guidelines (Table 6). Among the 10 isolates of *K. pneumoniae*, most of the strains showed sensitivity to CZA, PIT, AT, AK and MRP. Among the 10 strains, 5 were considered as AmpC producers as they showed sensitivity to cefoxitin less than 18mm, 3 strains were ESBL produces because they were resistant to all cephalosporin generations including aztreonam and 3 strains were carbapenemase producers as they show resistant to meropenem antibiotic less than 23mm according to CLSI guidelines (Table 7). Among the 3 isolates of *Proteus species*, most of the strains showed sensitivity to CAC, PIT, AK and MRP. Among the 3 strains, 2 were considered as AmpC producers as they showed sensitivity to cefoxitin less than 18mm, 2 strains were ESBL produces because they were resistant to all cephalosporin generations including aztreonam and one strain was carbapenemase producers as they show resistant to meropenem antibiotic less than 23mm according to CLSI guidelines (Table 8). Among the 2 isolates of *P. aeruginosa*, all were sensitive to TOB and showed AmpC ESBL and carbapenemase production (Table 9). Among the five *S. aureus* isolates, all they showed sensitivity to linezolid *i.e.* the zone size is greater than 26mm and 4 strains were sensitive to clindamycin. Among the 5 isolates, 2 were MRSA and 3 were MSSA. All the 2 MRSA strains were showing resistance to cefoxitn less than 22mm zone size (Table 10).

## Discussion:

One of the most prevalent cardiovascular conditions, stroke is influenced by underlying risk factors such as diabetes mellitus, high blood pressure, smoking, high cholesterol, atrial fibrillation and atherosclerosis, in addition to personal traits like age, gender and family history [22]. Understanding the role of both acute and chronic infections in stroke has drawn increased attention in recent decades. Fatty plaques in blood vessels, atherogenic reactions and altered host metabolism are all consequences of infection-induced inflammation [23]. The present study included a total of 60 patients and among them 63.3 % males and 36.6% were females. The mean age of the patients was 67.8 years. Alshaimaa *et al.* [24] in the year 2024 conducted a similar type of study to evaluate the predictors of stroke-associated pneumonia (SAP) in 520 ischemic stroke patients. The mean age group of the affected population was identified as 55 ± 10 years. In most of the study subjects of the current study, the major predisposing factors were Diabetes 46.7% followed by Hypertension 31.7%. In most of the epidemiologic studies, the authors showed that diabetes is a well-established independent but modifiable risk factor for stroke, both ischemic and hemorrhagic stroke (Chen *et al.* 2016) [26]. In majority of the acute stroke patients, most of the infection identified was UTI (48.3 %) followed by lower respiratory tract infections 33.3%. Alshaimaa *et al.* [24] reported out of 169 SAP cases, 9 (5.3%) cases were reported with bacteremia. Among the 169 SAP confirmed cases, 11 deaths were noted and no deaths from the non-SAP patients (P< 0.001). The authors concluded that, SAP was reported among one-third of the cases reported with ischemic heart disease and found that the major predictors of SAP were bulbar dysfunction, mechanical ventilation usage and prior history of stroke/transient ischemic stroke. Strict attention is needed for these vulnerable populations in order to reduce the risk of mortality. Among the 60 samples received, 46 samples were culture positive. The common microorganism isolated was *E.coli* (33.3%) followed by *Klebsiella pneumoniae* (16.6%), *Staphylococcus aureus* (8.3%). Among the 520 admitted patients, SAP was detected in 169 (32.4%) stroke patients. The most common microbial isolate from these patients was *Klebsiella pneumoniae* with a prevalence of 40.2%, followed by *Pseudomonas aeruginosa* (20.7%). Similar study was conducted by Rinawati *et al.* (2024) [25] studied the microbial profile of infectious patients suffered with acute ischemic stroke during Covid-19 period. The author conducted a retrospective study on 519 patients and noticed that, the prevalence of bacterial infections after acute ischemic stroke was 17.9%. The prevalence of bacterial infection following acute ischemic stroke was significantly different before and during the COVID-19 pandemic (87.5% vs. 10.8%). The most common bacteria found were Staphylococcus and Klebsiella sp. The risk factors that affected bacterial infection following acute ischemic stroke were sepsis, intensive care stay, COVID-19 infection, use of steroids *etc.* [25]. Ahmad *et al.* (2025) suggests that aerobic Gram-negative bacilli (*e.g.*, *K. pneumoniae*, *E. coli* and *P. aeruginosa*) and Gram-positive *cocci* (*e.g.*, *S. aureus* and *S. pneumoniae*) were associated with the majority of pneumonia complicating stroke [27]. Ji *et al.* [28] used data from a national registry study that included 14,702 stroke patients and discovered that pneumonia was linked to a two-fold higher risk of recurrent stroke in AIS patients while they were in the hospital (median length of stay, 14 days). Erdur *et al.* [29] discovered that pneumonia was not a risk factor for in-hospital stroke recurrence in all patients in retrospective research involving 5106 patients who had either an ischemic stroke or a transient ischemic attack. However, in a sensitivity analysis, pneumonia was linked to a 4-fold higher risk of recurrent stroke during hospitalization (median length of stay, 5 days) when the research participants were limited to those with transient ischemic attack or mild stroke (NIHSS score, ≤5). However in the present research, the average NIHSS score identified was 10.2 ± 3.8 indicating moderate stroke severities. This suggested that the veiling effect could be the cause, as it was more challenging to identify stroke recurrence in individuals who were more seriously afflicted. There are a number of possible explanations for why acute infection after stroke was linked to a higher risk of recurrent stroke in the early stages: cerebral ischemic events may result from procoagulant state, impaired fibrinolysis, hypoperfusion after peripheral circulatory failure, and inflammatory cascades following infection. Infection did not, however, have a long-term effect on stroke recurrence, suggesting that its influence on recurrence may wane with time. In the present research, most of the isolates showed good sensitivity to cephalosporins, carbapenems, macrolides, monobactams and cefamycins. However some strains showed the production of AMPC ESBL and carbapenemase production. In such cases, the second line antibiotics like Linezolid, Colistin, CZA +AT was used as a treatment option. According to Smith *et al.* (2019) stroke-associated pneumonia is predominantly associated with aerobic Gram-negative bacilli (*e.g.*, *Klebsiella pneumoniae* and Escherichia coli) and Gram-positive *cocci* (*e.g.*, Stapylococcus spp.) [30]. although the antibacterial spectrum of β-lactam antibiotics varies, third-generation cephalosporins and penicillin plus β-lactamase inhibitors are generally equivalent, even though their coverage of Pseudomonas species differs. On the other hand, macrolides have little effect on Gram-negative organisms and are mostly effective against Gram-positive ones (such as *Streptococcus pneumonia*e). Therefore, it doesn't appear likely that the antimicrobial spectrum would account for any positive effects of macrolide therapy for infections linked to stroke, particularly stroke-associated pneumonia. Based on the findings of the present study, it was suggested that, regular screening for infections in stroke patients through clinical observation and microbiological testing (*e.g.*, cultures, blood tests) can help in early treatment. In addition, implementing best practices for rehabilitation, including mobility support, nutrition, and skin care, can lower the risk of secondary infections.

## Conclusion:

Major infections in AIS patients has disclosed predominant causative pathogens in urinary tract infections, lower respiratory tract infections, blood stream and their susceptibility patterns to antibiotics which are needed for better management of patients and to mitigate mortality. Public health initiatives can focus on strategies to reduce infections in stroke patients, such as improving early detection of infections, optimizing the management of risk factors like aspiration and catheter use, and promoting hygiene and infection control practices.

## Figures and Tables

**Table 1:** Predisposing factors for developing microbial infections in acute ischemic stroke patients

**Predisposing factors**	**Frequency**	**Percentage**
Atherosclerosis	6	10%
Diabetes	28	46.70%
Hypertension	19	31.70%
Obesity	7	11.70%

**Table 2:** Samples collected from acute ischemic stroke patients

**Samples**	**Frequency**	**Percentage**
Blood	11	18.30%
Sputum	20	33.30%
Urine	29	48.30%

**Table 3:** Microorganisms isolated

**Microorganisms isolated**	**Frequency**	**Percentage**
Total sample size	60	100%
No growth	14	23.30%
Culture positive	46	76.60%
*E. coli*	20	33.30%
*Klebsiella pneumoniae*	10	16.60%
*Acinetobacter baumanii*	3	5%
*Streptococcus species*	2	3.30%
*Proteus species*	4	6.60%
*Pseudomonas aeruginosa*	2	3.30%
*Staphylococcus aureus*	5	8.30%

**Table 4:** Antibiotic susceptibility pattern of *Acinetobacter baumanii*

**Antibiotics**	**Number of isolates sensitive to antibiotics**	**Number of isolates resistant to antibiotics**
COT	1	2
CZA	3	Nil
CTR	1	2
AMC	Nil	3
CAZ	1	2
CX	Nil	3
CAC	1	2
AT	1	2
CPM	1	2
PIT	2	1
AK	2	1
CTX	1	2
TE	2	1
MRP	1	2
A/S	1	2
Total number of AmpC producers	3	
Total number of ESBL producers	2	
Total of Carbapenemase producer	2	

**Table 5:** Antibiotic susceptibility pattern of *Streptococcus pneumoniae*

**Antibiotics**	**Number of isolates sensitive to antibiotics**	**Number of isolates resistant to antibiotics**
Sensitive to Bacitracin	Nil	2
Sensitive to Optochin	2	Nil
P	Nil	2
AMX	Nil	2
LVX	2	Nil
CTX	1	1
CTR	1	1
COT	1	1
VAN	2	Nil
MRP	2	Nil
E	2	Nil
TE	1	1
CD	2	Nil
DOX	2	Nil
CPM	2	Nil
AMC	1	1
CXM	1	1
LZ	2	Nil

**Table 6:** Antibiotic susceptibility pattern of *E.coli*

**Antibiotics**	**Number of isolates sensitive to antibiotics**	**Number of isolates resistant to antibiotics**
COT	3	17
CZA	15	5
CTR	8	12
AMC	5	15
CAZ	8	12
CX	14	6
CAC	13	7
AT	8	12
CPM	8	12
PIT	14	6
AK	18	2
TE	5	15
MRP	18	2
GEN	11	9
CTX	8	12
Total number of AmpC producers	6	
Total number of ESBL producers	12	
Total of Carbapenemase producer	2	

**Table 7:** Antibiotic susceptibility pattern of *Klebsiella pneumoniae*

**Antibiotics**	**Number of isolates sensitive to antibiotics**	**Number of isolates resistant to antibiotics**
COT	2	8
CZA	7	3
CTR	3	7
AMC	1	9
CAZ	3	7
CX	5	5
CAC	5	5
AT	8	2
CPM	3	7
PIT	6	4
AK	6	4
TE	4	6
MRP	7	3
GEN	4	6
CTX	3	7
Total number of AmpC producers	5	
Total number of ESBL producers	3	
Total of Carbapenemase producer	3	

**Table 8:** Antibiotic susceptibility pattern of *Proteus species*

**Antibiotics**	**Number of isolates sensitive to antibiotics**	**Number of isolates resistant to antibiotics**
COT	1	3
CZA	2	2
CTR	2	2
AMC	1	3
CAZ	2	2
CX	2	2
CAC	3	1
AT	2	2
CPM	2	2
PIT	3	1
AK	3	1
TE	1	3
MRP	3	1
GEN	2	2
CTX	2	2
Total number of AmpC producers	2	
Total number of ESBL producers	2	
Total of Carbapenemase producer	1	

**Table 9:** Antibiotic susceptibility pattern of *Pseudomonas aeruginosa*

**Antibiotics**	**Number of isolates sensitive to antibiotics**	**Number of isolates resistant to antibiotics**
CAZ	Nil	2
CX	Nil	2
CAC	1	1
TOB	2	Nil
CPM	Nil	2
PIT	1	1
MRP	Nil	2
PI	1	1
TE	1	1
GEN	Nil	2
CIP	Nil	2
Total number of AmpC producers	2	
Total number of ESBL producers	2	
Total of Carbapenemase producer	2	

**Table 10:** Antibiotic susceptibility pattern of *Staphylococcus aureus*

**Antibiotics**	**Number of isolates sensitive to antibiotics**	**Number of isolates resistant to antibiotics**
CX	3 (MSSA)	2 (MRSA)
LZ	5	Nil
GEN	2	3
E	3	2
CD	4	1
TE	2	3
CIP	1	4
COT	1	4
P	Nil	5

## References

[1] Feigin V.L (2014). Lancet..

[2] GBD 2017 Causes of Death Collaborators. (2018). Lancet..

[3] Horton R, Das P (2011). Lancet..

[4] Yamunadevi A, Sulaja S (2016). Int J Asian Social Sci..

[5] Dalal P (2007). Ann Ind Acad Neurol..

[6] Naik K.R (2016). Ind J Health Sci Biomed Res (KLEU)..

[7] https://nhm.gov.in/.

[8] Kamalakannan S (2017). Ind J Med Res..

[9] Keikha M, Karbalaei M (2022). New Microbes New Infect..

[10] Lucchese G (2019). Front Neurol..

[11] Budzynski J (2014). Clin Res Cardiol..

[12] Sessa R (2014). Clin Case: WJCC..

[13] Sessa R (2014). World J Clin Case: WJCC..

[14] Chen B.F (2013). Helicobacter..

[15] Chen J (2013). BMC Neurol..

[16] Chen Y (2008). Europ J Vasc Endovasc Surg..

[17] McCaughey C (2005). BMC Infect Dise..

[18] Keikha M (2020). Virusdisease..

[19] García-Cabrera E (2013). Circulation..

[20] Wasay M (2018). Stroke..

[21] Van de Beek D (2009). Arch Neurol..

[22] Simanek A.M (2011). PloS One..

[23] Fugate J.E (2014). Lancet Infect Dise..

[24] Aboulfotooh A.M (2024). BMC Neurol..

[25] Rinawati W (2024). Medicina (Kaunas)..

[26] Chen R (2016). Am J Med Sci..

[27] Abujaber A.A (2025). Journal of Stroke and Cerebrovascular Diseases..

[28] Ji R (2013). Stroke..

[29] Erdur H (2015). Stroke..

[30] Smith C.J (2019). Front Neurol..

